# The Methodology for Evaluating the Operating State of SF6 HVCBs Based on IDDA

**DOI:** 10.3390/s24082513

**Published:** 2024-04-14

**Authors:** Tong Bai, Chenhao Sun, Wenqing Feng, Yajing Liu, Huanzhen Zhang, Yujia Wang

**Affiliations:** 1 School of Electrical & Information Engineering, Changsha University of Science & Technology, Changsha 410004, China; btxiaoying@gmail.com (T.B.); 18978088097@163.com (Y.L.); 13975135009@163.com (H.Z.); 2 Computer & Software School, Hangzhou Dianzi University, Hangzhou 310018, China; wq_feng@hdu.edu.cn; 3 School of International College of Engineering, Changsha University of Science & Technology, Changsha 410004, China; wangyujia0724@outlook.com

**Keywords:** SF6 HVCBs, data-driven analysis, critical factor stability, fuzzy inference system

## Abstract

To enhance the precision of evaluating the operational status of SF6 high-voltage circuit breakers (HVCBs) and devise judicious maintenance strategies, this study introduces an operational state assessment method for SF6 HVCBs grounded in the integrated data-driven analysis (IDDA) model. The relative degradation weight (RDW) is introduced as a metric for quantifying the relative significance of distinct indicators concerning the operational condition of SF6 HVCBs. A data-driven model, founded on critical factor stability (CFS), is formulated to convert environmental indicators into quantitative computations. Furthermore, an optimized fuzzy inference (OFI) system is devised to streamline the system architecture and enhance the processing speed of continuous indicators. Ultimately, the efficacy of the proposed model is substantiated through validation, and results from instance analyses underscore that the presented approach not only attains heightened accuracy in assessment compared to extant analytical methodologies but also furnishes a dependable foundation for prioritizing maintenance sequences across diverse components.

## 1. Introduction

SF6 high-voltage circuit breakers (HVCBs) constitute essential electrical switching devices within power systems, concurrently fulfilling the critical functions of circuit interruption, closure, and provision of control and protective measures [1,2,3]. Comprehensively assessing the health status of SF6 HVCBs is a prerequisite for determining their safety control levels and implementing equipment maintenance strategies.

Currently, numerous researchers have proposed an abundance of methods for evaluating the operating state of HVCBs, yielding substantial achievements. These methods can be primarily categorized into the following two types.

The first classification pertains to conventional techniques of measurement and surveillance, which leverage sensors and instrumentation for the real-time monitoring of circuit breaker parameters. Faults within HVCBs are identified through the analysis of parameters such as electrical or vibration signals. For example, in reference [4], both mechanical and electrical signals are gathered and subjected to analysis, culminating in the proposal of a fault diagnosis methodology for HVCBs predicated on the fusion of multisensory information. Ref. [5] introduces an enhanced rule for the combination of multi-sensor evidence aimed at optimizing the data acquired from diverse sensors. Qiuyu Yang et al. present a method for assessing the state of spring-operated HVCB dampers based on vibration time frequency imagery [6]. Ref. [7] employs the characteristics of control coil currents as modeling data and advances an online amalgamated HVCB fault diagnosis approach, thereby augmenting diagnostic precision and the capacity for learning.

The second category of methods involves the integration of intelligent algorithms with data-driven approaches to evaluate the health status of circuit breakers [8]. Data-driven analysis, based on the degree of correlation between data, integrates technologies such as data mining, extensive storage, fuzzy mathematics, expert systems, and machine learning to establish empirical models. These models combine a wealth of data with the actual operating conditions of the equipment to model and predict the operating state of circuit breakers. In Ref. [9], to extract and simulate the correlation between circuit breaker characteristic indicators and operating conditions, an adaptive error back propagation neural network (BPNN) was constructed with parameter improvements. Yao et al. employed a combination of fractal technology and probabilistic neural network for a circuit breaker fault diagnosis method capable of classifying and identifying faults [10]. Advanced artificial intelligence methods, including fuzzy logic, k-means clustering, cluster trees, and artificial neural networks, were introduced in [11] to improve the health assessment of HVCBs. Ref. [12] proposes a comprehensive diagnosis of mechanical faults in HVCBs using a multi-channel integrated convolutional neural network based on multi-data fusion. Diahovchenko et al. developed a fuzzy logic-based assessment method for SF6 HVCBs, determining the equipment most in need of maintenance and assisting in prioritizing maintenance plans [13].

While the scholarly endeavors mentioned above have achieved significant advancements, certain areas persist that necessitate refinement. Firstly, some methodologies exhibit a lack of comprehensive consideration for evaluation factors, often resulting in the exclusion of environmental data from the assessment model due to challenges in quantifying the influence of environmental indicators on circuit breaker operational status. Secondly, the unequal impact of varying factors on evaluation outcomes remains inadequately addressed. Lastly, when dealing with input indicators featuring both discrete and continuous data, prevailing analytical approaches frequently rely on a uniform data-driven model, potentially overlooking the intrinsic disparities between continuous and discrete data types, thereby impacting the precision of the evaluation model.

In response to these challenges, this study proposes an operational status assessment methodology for SF6 HVCBs predicated on the integrated data-driven analysis (IDDA) model. Initially, a comprehensive database is established, comprising fault precursor indicators of diverse data types that encapsulate the mechanical, electrical, and insulation performance of the circuit breaker, alongside environmental attributes. Subsequently, to mitigate the imbalances induced by variations in distinct factors affecting system stability, a data-driven model grounded in critical factor stability (CFS) is deployed to objectively assess the relative significance of environmental factors. Lastly, an optimized fuzzy inference (OFI) system, integrated with probabilistic fuzzy and hierarchical fuzzy techniques, is employed to process continuous numerical indicators, thus streamlining the complexity of the model. The results of the case analysis indicate that the proposed SF6 HVCB operational status assessment method can enhance the accuracy of the evaluation.

## 2. Data Preprocessing

### 2.1. Data Collection

The operational stability of SF6 HVCBs relies heavily on the mechanical, electrical, and insulation performances. A comprehensive assessment of SF6 HVCBs’ operational condition necessitates the consideration of these three key performances. Currently, the primary focus of circuit breaker monitoring includes dynamic contact travel, coil current, vibration signals, stored energy motor current, and various other parameters [14]. Given the multitude of signals available for safety assessment, selected signals should exhibit the following characteristics:

1. They should accurately represent variations in different components or operating states of the circuit breaker.
2. They should promptly detect changes in the equipment’s status.
3. They should furnish precise and dependable monitoring data.
4. They should ease measurement and analysis.

We amalgamated the aforementioned characteristics and extensively consulted experts and on-site operators to gather the following signal data.

Mechanical performance primarily pertains to the efficacy of mechanical structures such as the opening and closing mechanism, contact system, and mechanical connectors, which reflect operational processes and mechanical status. An SF6 HVCB characterized by abbreviated opening and closing durations and rapid operational velocity facilitates expeditious disconnection and reconnection of electrical circuits. Measurement of opening and closing time and speed enables the assessment of spring and operating mechanism sensitivity and reliability. Contact travel directly impacts the circuit breaker’s contact state and arc formation. Excessive contact travel may augment wear and energy loss. Hence, measuring contact travel and ensuring it falls within an appropriate range are imperative to mitigate issues such as contact wear and poor contact.

Electrical performance encompasses the breaking capacity of SF6 HVCBs, stability of the control system, and reliability of electrical connections. Breaking current value is gauged through a current transformer to characterize circuit breaker breaking performance, reflecting remaining life and relative contact wear. Operating current and voltage values of opening and closing coils ascertain the condition of coil magnet operation and control circuit functionality, providing insights into coil status and locking device operation.

SF6 gas serves as the arc extinguishing medium for circuit breakers, with its insulation performance directly impacting safety and stability. To evaluate the insulation capability and arc extinguishing performance of SF6 HVCBs, temperature, pressure, density, and leakage of SF6 gas must be measured. Insufficient gas pressure or density decrease may result in local discharge of the arc extinguishing medium.

Environmental factors indirectly affect insulation, electrical, and mechanical performance within SF6 HVCBs, thereby influencing operational status. Therefore, recording surrounding environmental conditions is essential. Additionally, according to the equipment ledger and maintenance records, collect records of operational conditions.

### 2.2. Data Categorization

The data collected has been categorized into discrete and continuous indicators. Discrete indicators predominantly pertain to environmental variables such as weather, pollutants, mechanical vibration, external physical damage, and electromagnetic disturbances. Each indicator encompasses distinct factors, as delineated in Table 1.

Continuous indicators encompass mechanical, electrical, and insulation performance metrics. These metrics, along with their associated safety thresholds [11], are detailed in Table 2. Should a specific indicator surpass its safety threshold, it is deemed a rare factor, potentially resulting in irregular equipment conditions.

Opening and closing time (ms$U_{o}=(0.65,1.1)U_{n}$$U_{c}=(0.85,1.1)U_{n}$$U_{o}/R$$U_{c}/R$ Notably, SF6 HVCBs of different rated voltage levels have varying opening and closing coil voltages. $U_{n}$ signifies the rated voltage of SF6 HVCBs, $U_{o}$ denotes the voltage of the opening coil, $U_{c}$ represents the voltage of the closing coil, and R denotes the resistance value of the measured coil.

The operational status of SF6 HVCBs based on events is categorized into four levels: Excellent, Fine, Abnormal, and Severe. Different levels correspond to different treatment methods. The corresponding set of comments is presented in Table 3.

### 2.3. Establishment of the Data Matrix

In order to facilitate subsequent data-driven analysis, all input data undergo preprocessing to form a data processing space. Considering that the collected data of continuous indicators such as opening and closing time, speed, and total contact travel involve multiple scales and dimensions, it is necessary to first normalize the value of indicators. The formula is as follows:

(1) $$e_{j,p}^{*}=\frac{e_{j,p}-e_{min}}{e_{max}-e_{min}}.$$

Here, $e_{j,p}$ represents any one of the indicator values in indicator $i_{j}$, where $e_{max}$ and $e_{min}$, respectively, denote the maximum and minimum values of the indicator, and $e_{j,p}^{*}$ is the normalized indicator value.

Then, let $\left\{r_{1},r_{2},r_{3},\ldots ,r_{m}\right\}$ be a set containing the identifiers of each status record, and let $\left\{i_{1},i_{2},i_{3},{\ldots ,i}_{j},\ldots ,i_{17}\right\}$ be a group containing 17 indicators, where each indicator $i_{j}$ is composed of a set of indicator factors, $e_{j,1},e_{j,2},\ldots ,e_{j,p},\ldots e_{j,w}$. Let $\left\{V_{1},V_{2},V_{3},V_{4}\right\}$ be a group containing four operational status ratings. To handle discrete and continuous indicators separately, the set containing indicators is divided into two parts and represented in matrix form, where discrete indicator factors are denoted as $I_{d}$, and continuous indicator factors are denoted as $I_{c}$:

(2) $$I_{d}=\left[i_{1},i_{2},i_{3},i_{4},i_{5}\right],$$

(3) $$I_{c}=\left[i_{6},\ldots ,i_{15},i_{17}\right].$$

Based on the above assumptions, an integrated database space matrix, denoted as M, can be constructed:

(4) $$M=\left[\begin{array}{c} \begin{array}{ccccccc} R & i_{1} & \cdots & i_{j} & \cdots & i_{n} & V\\ r_{1} & e_{11} & \cdots & e_{1j} & \cdots & e_{1n} & V_{1}\\ r_{2} & e_{21} & \cdots & e_{2j} & \cdots & e_{2n} & V_{2}\\ \vdots & \vdots & \ddots & \vdots & \ddots & \vdots & \vdots \\ r_{i} & e_{i1} & \cdots & e_{ij} & \cdots & e_{in} & V_{i}\\ \vdots & \vdots & \ddots & \vdots & \ddots & \vdots & \vdots \\ r_{m} & e_{m1} & \cdots & e_{mj} & \cdots & e_{mn} & V_{m} \end{array} \end{array}\right].$$

Here, $e_{ij}$ represents one factor of indicator $i_{j}$ recorded in status record $r_{i}$, corresponding to any factor $e_{j,p}$ in the respective column; $V_{i}$ is the multi-factor-determined operational status rating of the SF6 HVCB, where $V_{i}\in \{V_{1},V_{2},V_{3},V_{4}\}$.

## 3. Establishment of the IDDA Analysis Model

### 3.1. Definition of Relative Degradation Weight

We propose the relative degradation weight (RDW) to represent the impact of various factors on the operational state of circuit breakers, where a lower score indicates a more stable operational state. The score of the RDW for the SF6 HVCB is represented by the following equation:

(5) $$\Psi_{HVCB}=\sum_{a}^{M}\Psi_{e_{j,p}}^{d}+\sum_{b}^{N}\Psi_{e_{j,p}}^{c}.$$

Here, $\Psi_{HVCB}$ represents the RDW for the SF6 HVCB and $\Psi_{e_{j,p}}^{d}$ and $\Psi_{e_{j,p}}^{c}$, respectively, denote the RDW for the a-th discrete indicator and the b-th continuous indicator. M and N represent the numbers of discrete and continuous indicators, respectively.

After obtaining the value of $\Psi_{HVBC}$, it needs to be normalized using the following formula:

(6) $$\Psi_{HVCB}^{*}=\frac{\Psi_{HVCB}}{10^{d}}.$$

where d is the number of digits that makes the absolute maximum value of $\Psi_{HVCB}$. The ranges of $\Psi_{HVCB}^{*}$ corresponding to different operational conditions are as follows: Excellent: (0, 0.25), Fine: (0.25, 0.50), Abnormal: (0.50, 0.75), Severe: (0.75, 1.00).

### 3.2. Data-Driven Analysis Model Based on CFS

#### 3.2.1. The Background of CFS Analysis

CFS analysis pertains to the examination of pivotal elements influencing the stability of a system. Rooted in the theoretical framework of system stability, the collective stability of a system is predominantly contingent upon the stability exhibited by its constituent components. However, it is acknowledged that not all components contribute uniformly to sustaining system stability [15]. Enhancing the stability of specific critical components holds the potential to exert a substantial positive influence on the overall stability of the system. Moreover, given the constraints of human and material resources, the identification of critical components becomes imperative, guiding the judicious allocation of resources towards their vigilant monitoring and maintenance [16].

The process of importance measurement facilitates the discernment of the relative significance of components in relation to others within the system [17]. In the context of this study, components encapsulate diverse factors encompassed within each indicator. Through the application of importance measurement, the discernible impact of distinct factors on overall stability is elucidated, thereby facilitating the identification of pivotal factors that significantly influence system stability.

#### 3.2.2. Identification and Selection of RP Factors

According to the definition of association rules [18], let $E=\{e_{1},e_{2},e_{3}...\}$ be the input database. Assuming sets U and V satisfy U⊂E,V⊂E, U⋂V=∅ and meet the condition expressed as U⇒V, the occurrence of events U and V is considered to be correlated. Here, U represents the set of factors and V represents the set of comments. Further dividing the factor set into a frequent factor set and a rare factor set for analysis, a correlated event can be expanded into two parts:

(7) $$U^{f}+U^{r}\Rightarrow V,$$

where $U^{f}$ and $U^{r}$ represent the frequent factor set and rare factor set, respectively.

The deterioration of certain rare factors may lead to a drastic decline in the operational stability of the circuit breaker. While these factors do not occur frequently, they have a critical impact on the system and should be considered as crucial factors. We refer to them as rare but pivotal (RP) factors. RP factors are often discarded because their diagnostic scores do not meet the set threshold. Currently, common diagnostic criteria include support and confidence [19]. Therefore, this section adopts an improved diagnostic criterion calculation method to calculate the scores of rare factors. When the score exceeds the set threshold, the factor is considered an RP factor. If an association event, $U^{f}+U^{r}\Rightarrow V$, includes any rare factor associated with an indicator, $i_{j}$, their support and confidence can be expressed by the following equations:

(8) $$H_{\left(supp\right)j}=\frac{Num\left|r_{i}\in M\left(i,1\right);U^{f}\subseteq M\left(i,Z_{g}\right)\neq \emptyset ;M\left(i,j\right)\in U^{r}\neq \emptyset \right|}{Num\left|r_{i}\in M\left(i,1\right);M\left(i,j\right)\in U^{r}\neq \emptyset \right|},$$

(9) $$H_{\left(conf\right)j}=\frac{Num\left|r_{i}\in M\left(i,1\right);U^{f}\subseteq M\left(i,Z_{g}\right)\neq \emptyset ;M\left(i,j\right)\in U^{r}\neq \emptyset ;M\left(i,n+2\right)=V\left(o\right)\right|}{Num\left|r_{i}\in M\left(i,1\right);M\left(i,j\right)\in U^{r}\neq \emptyset \right|}.$$

In these equations, $H_{(supp)j}$ and $H_{(conf)j}$ represent the improved support and confidence, respectively. Num|·| represents the cardinality of the state records in M that simultaneously satisfy all included conditions. $Z_{g}$ represents the numerical range from 2 to (n+1), and V(o) represents the operating state of the SF6 HVCB.

#### 3.2.3. The Resolution of RDW Based on CFS

We define $G^{RDW}$ as the relative decrease in the stability of the circuit breaker operating system when the factor $e_{j,p}$ is absent [20], and its mathematical expression is as follows:

(10) $$G^{RDW}\left(e_{j,p}|r_{i}\right)=\frac{1-g\left(f\left(r_{i}\right)\right)}{1-g\left(0_{k},f\left(r_{i}\right)\right)}.$$

Here, $1-g(f(r_{i}))$ represents the stability of fault $r_{i}$ occurring in the system and $1-g(0_{k},f(r_{i}))$ represents the stability of fault $r_{i}$ occurring in the system when the factor $e_{j,p}$ is confirmed not to be present.

To analyze the frequent variable set and the rare variable set separately, in this chapter, we define a submatrix $M_{rp}$ of matrix M. $M_{rp}$ comprises state records of any RP factor within the indicator $i_{j}$. Additionally, based on the set of factors included in a single performance indicator $i_{j}$, this section constructs the $i_{jf}$ (frequent factor subset) and $i_{jr}$ (rare factor subset).

In a real operational state record, there is a sequential relationship among various environmental indicators. Based on this, the overall stability of the system can be determined by the product of the comprehensive likelihood of abnormal states occurring in the system when the corresponding factors appear in each indicator. The mathematical expression is:

(11) $$1-g\left(f\left(r_{i}\right)\right)=1-\prod_{j=2}^{n+1}g\left(0_{k},f\left(r_{i}\right)\right)=1-\prod_{j=2}^{n+1}\left(\sum_{i=2}^{Num\left|r_{i}\in M_{rp}\right|}\frac{Num\left|r_{i}\in M\left(i,1\right);M\left(i,j\right)=e_{j,p};M\left(i,j\right)\in i_{jr}\right|}{Num\left|r_{i}\in M\left(i,1\right);M\left(i,j\right)\in i_{j}\right|}\right).$$

We define the relative deterioration weight, $\Psi_{e_{j,p}}$, for an individual factor, $\;e_{j,p}\in i_{j}$. The RDW consists of two components and can be expressed as:

(12) $$\Psi_{e_{j,p}}^{d}=\Psi_{j,p}^{f}+\Psi_{j,p}^{r}.$$

In this equation, $\Psi_{j,p}^{f}$ represents the RDW of frequent factors and $\Psi_{j,p}^{r}$ represents the RDW from rare factors. The mathematical expression for $\Psi_{j,p}^{f}$ can be written as:

(13) $$\Psi_{j,p}^{f}=\left\{\begin{array}{c} \sum_{i=2}^{Num\left|r_{i}\in M\right|}\frac{Num\left|M\left(i,j\right)=e_{j,p}\right|}{Num\left|m\right|},e_{j,p}\in i_{jf}\\ 0\;,\;e_{j,p}\in i_{jr} \end{array}\right..$$

Here, i=2,3,…,(m+1) represents an operational record in the data space matrix M, j=2,3,…,(n+1) represents an environmental indicator $i_{j}$, and Num|m| represents the cardinality of all fault records in M. Combining the Equation (8) with the Equation (9), the mathematical expression for $\Psi_{j,p}^{r}$ in $\Psi_{e_{j,p}}$ is:

(14) $$\Psi_{j,p}^{r}=\left\{\begin{array}{c} \frac{1-\prod_{j=2}^{n+1}\left(\sum_{i=2}^{Num\left|r_{i}\in M_{rp}\right|}\frac{Num\left|r_{i}\in M\left(i,1\right);M\left(i,j\right)=e_{j,p};M\left(i,j\right)\in i_{jr}\right|}{Num\left|r_{i}\in M\left(I,1\right);M\left(I,j\right)\in i_{j}\right|}\right)}{1-\prod_{P=1}^{w}\left(\sum_{i=2}^{Num\left|r_{i}\in M_{rp}\right|}\frac{Num\left|r_{i}\in M\left(i,1\right);M\left(i,j\right)\neq e_{j,p};M\left(i,j\right)\in i_{jr}\right|}{Num\left|r_{i}\in M\left(i,1\right);M\left(i,j\right)\in i_{j}\right|}\right)},\;e_{j,p}\in i_{jr}\\ 0,\;e_{j,p}\in i_{jf} \end{array}\right..$$

### 3.3. Data-Driven Model Based on the OFI System

#### 3.3.1. The Background of OFI System

Lotfi Zadeh introduced the term “fuzzy logic” in 1965 [21], and since then, the growing interest in fuzzy logic has led to the emergence of different branches [22]. In our work, we leverage classical fuzzy inference (FI), probabilistic fuzzy logic, and hierarchical fuzzy inference to propose an OFI system designed for calculating the RDW of continuous indicators.

**Figure 1:**
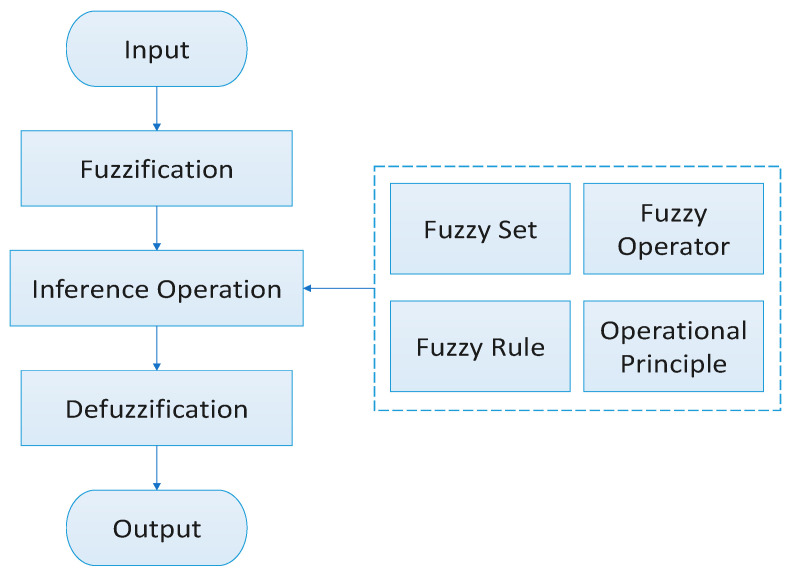
Fuzzy logic flowchart.

FI involves mapping based on fuzzy mathematics and membership theory. This allows the conversion of quantitative factors into a mathematically assessed state. In situations where the subject of study displays both fuzziness and continuity, classical logic alone may not provide an adequate description. For example, when defining the normal temperature range for SF6 gas as $\left(20.0\right.,47.3)$ °C and $\left(47.3\right.,83.2)$ °C as slightly high, determining whether a temperature value of 47 °C falls into the slightly high or normal range becomes challenging. Fuzzy logic, however, permits the membership of elements to a set to take any value in [0,1], making the boundaries between concepts unclear and facilitating continuous transitions between different parts. The typical flowchart of an FI system is illustrated in Figure 1, comprising three main modules: fuzzification, inference operation, and defuzzification.

To surmount the intricacies inherent in classical fuzzy logic when confronted with extensive and intricate datasets, we incorporate probabilistic fuzzy logic and hierarchical fuzzy logic into our devised FI system.

The probabilistic fuzzy methodology entails resolving the probability density distribution for each input indicator, normalizing the input indicators, and transforming them into probability values within the interval [0,1]. This approach is advantageous in that it relies solely on the frequency of occurrence of each indicator factor in the state records, facilitating a more straightforward analysis of RP factors. Furthermore, the utilization of the probabilistic fuzzy method obviates the necessity to establish distinct membership functions for individual indicators. Consequently, the proliferation of constructed membership functions is markedly curtailed, augmenting the system’s adaptability across diverse scenarios.

While the probabilistic fuzzy approach contributes to mitigating system complexity to some degree, the formulation of a comprehensive rule base for an extensive corpus of input data remains a formidable task. Consequently, our devised OFI system incorporates a hierarchical fuzzy inference approach for ascertaining the RDW of continuous indicators. This approach leverages the fuzzy output from the preceding layer as input for subsequent layers of fuzzy calculations [23].

**Figure 2:**
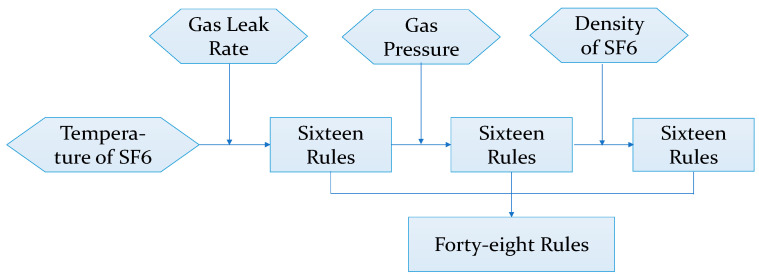
Structure of hierarchical fuzzy inference.

Illustratively, in the computation of the insulation performance indicator, employing the traditional Mamdani [24] fuzzy inference method for four continuous indicators, each containing four fuzzy sets, would necessitate 4^4^ fuzzy rules, imposing a substantial computational burden. Conversely, through hierarchical fuzzy inference, only 48 fuzzy rules suffice, effectively diminishing the computational load. The flowchart delineating the hierarchical fuzzy inference process is depicted in Figure 2.

#### 3.3.2. RDW Calculation Based on OFI

**Figure 3:**
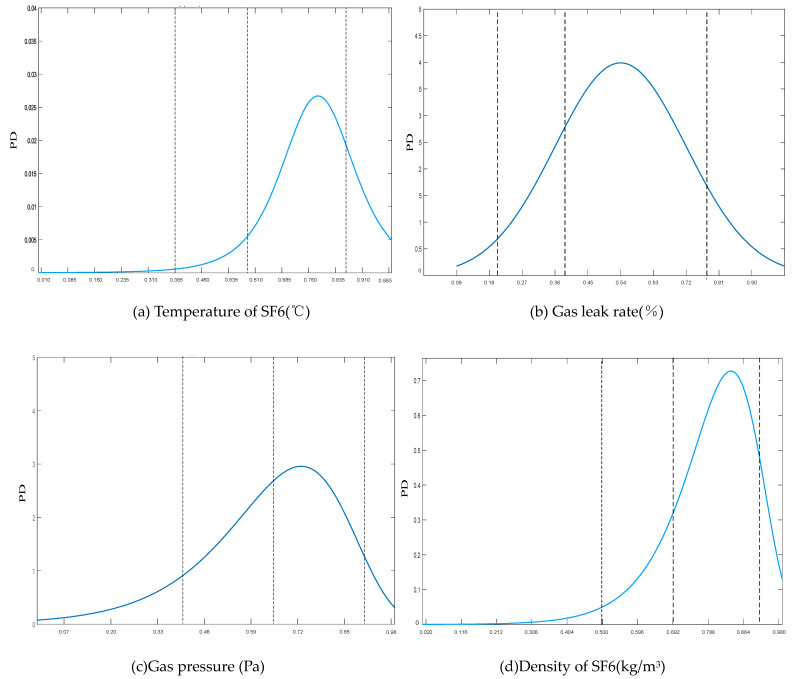
PDF of insulation performance indicators.

In the preliminary phase, the probability density function (PDF) curve is employed to ascertain the probability distribution of continuous indicators. The configuration of four fuzzy sets in both input and output membership functions, as outlined in this study, delineates the division of the PDF into four distinct numerical intervals. Each of these intervals corresponds to a specific fuzzy set. Taking the insulation performance indicators as an example, the PDF curves for the four indicators are shown in Figure 3. The quantification of the area bounded by the PDF curve within a given numerical interval, juxtaposed against the horizontal axis, serves to denote the probability associated with the continuous indicator falling within that particular fuzzy set.

The transformation of probabilities associated with resolving continuous indicator values, pertaining to each numerical interval, into distinct linguistic variables is accomplished through the utilization of membership functions. These functions serve as the foundational framework for the application of fuzzy sets to practical problem domains, with commonly employed analytical approaches encompassing trapezoidal distribution, triangular distribution, and Gaussian distribution. This study adopts the assignment method for the determination of both input and output membership functions.

**Figure 4:**
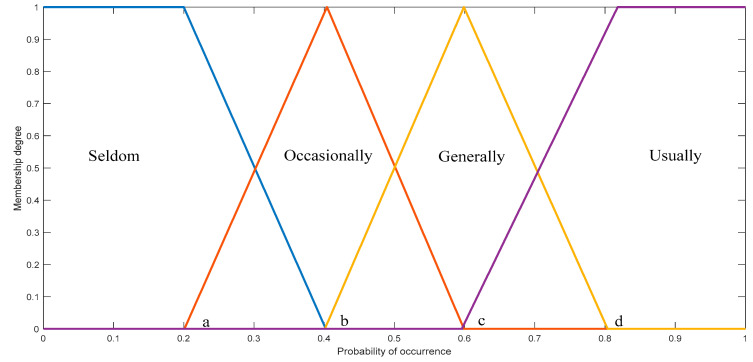
Input membership functions.

For quantifiable evaluation indicators such as mechanical performance, electrical performance, and insulation performance, a consistent combination of trapezoidal and triangular distributions is employed. The input membership functions for each continuous indicator share a uniform shape, differing solely at the boundaries of the fuzzy sets. As an illustrative instance, the input membership function for “SF6 temperature” is presented in Figure 4.

The delineation of the four regions within the input membership function is articulated as follows: Seldom, Occasionally, Generally, and Usually.

The membership function for “Seldom” is defined by the formula:

(15) $$f\left(e_{j,p}\right)=\left\{\begin{array}{c} 1,e_{j,p}\leq a\\ \frac{b-e_{j,p}}{b-a},a\leq e_{j,p}\leq b\\ 0,\mathrm{other} \end{array}\right..$$

The membership function for “Occasionally” is defined by the formula:

(16) $$f\left(e_{j,p}\right)=\left\{\begin{array}{c} \frac{e_{j,p}-a}{b-a},{a\leq e}_{j,p}\leq b\\ \frac{c-e_{j,p}}{c-b},b\leq e_{j,p}\leq c\\ 0,\mathrm{other} \end{array}\right..$$

The membership functions for the remaining regions can be obtained in a similar manner. Here, $e_{j,p}$ represents the p-th factor of indicator $i_{j}$, where j=1,2,3,…,n and p=1,2,3,…,w. a,b,c,d denote the boundary values of different fuzzy sets, and the boundary values for different input indicators are set based on practical considerations.

In accordance with the hierarchical fuzzy system structure, the formulation of the fuzzy rule set is delineated in Table 4 and Table 5. This study devises four distinct categories to characterize the contribution levels of the fuzzy relative deterioration degree, namely: Low, Moderate, Elevated, and Very High. Each category is associated with corresponding fuzzy weights of 0.15, 0.33, 0.58, and 0.89. It is noteworthy that factors occurring seldom are frequently indicative of highly unstable operating conditions, thereby aligning with a very high contribution level to the relative deterioration degree.

**Figure 5:**
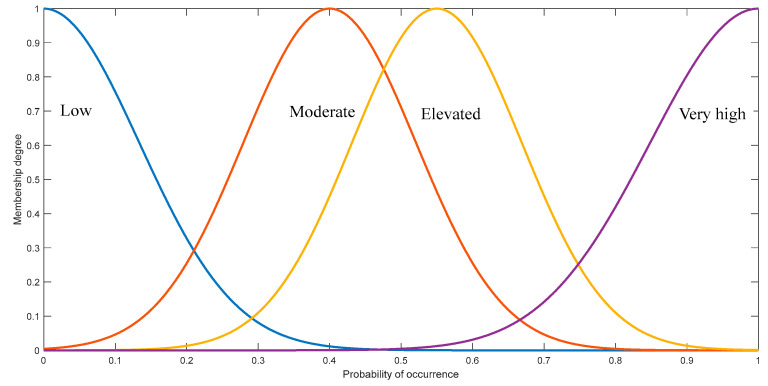
Output membership functions.

Finally, this section formulates the output membership functions based on four sets of Gaussian functions, as depicted in Figure 5. The designation of relative deterioration levels is established with means of 0 for “Low”, 0.4 for “Moderate”, 0.55 for “Elevated”, and 1 for “Very High”. Through the consolidation of all entries in the input database, the data distribution corresponding to deterioration levels is discerned. Subsequent to the weighting and aggregation of the determined probability regions, the cumulative regions undergo defuzzification using the centroid method to ascertain the RDW.

## 4. Operational State Evaluation Process

**Figure 6:**
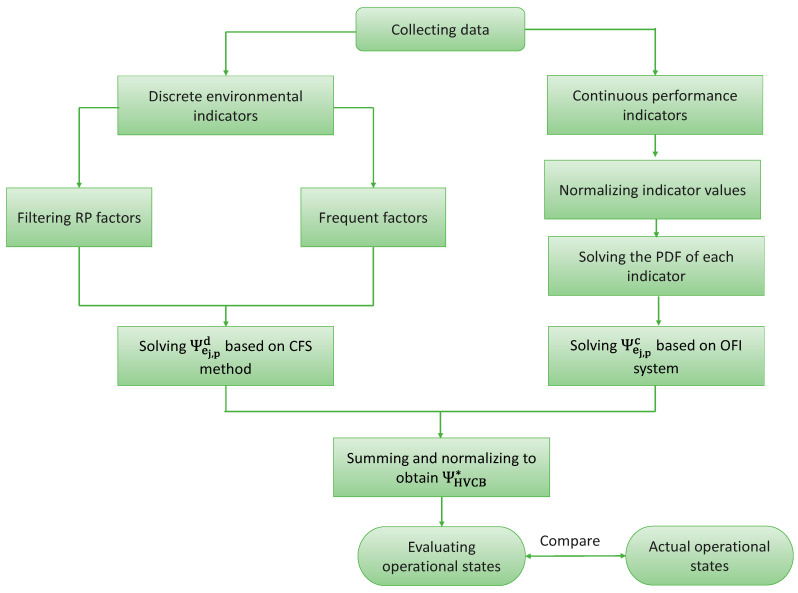
Flowchart.

According to the above discussion, we construct an IDDA-based operational state evaluation model for SF6 HVCBs. The flowchart of the basic process is illustrated in Figure 6.

The specific steps are as follows:

1. Collect data on environmental information, mechanical performance, electrical performance, and insulation performance and create a database;
2. Introduce the concept of RDW to quantify the operational state evaluation results of SF6 HVCBs;
3. Classify the data in the database into discrete and continuous indicators;
4. For discrete indicators, further explore and analyze the RP factors through an improved diagnostic criterion calculation method;
5. Repeat step 4 for each discrete indicator in the database, filter out the frequent factor set and the RP factor set, and calculate $\Psi_{e_{j,p}}^{d}$ using Formula (12);
6. For continuous performance indicators, normalize the data first;
7. Solve the PDF for continuous indicators;
8. Repeat step 7 for each continuous indicator in the database, and calculate $\Psi_{e_{j,p}}^{c}$ using a hierarchical fuzzy inference system;
9. Using Formulas (5) and (6) to calculate $\Psi_{HVCB}^{*}$, compare the final evaluation results with the true states recorded to assess the evaluation performance of the IDDA model.

## 5. Case Study

### 5.1. Test Data

We perform experimental validation utilizing recorded data derived from SF6 HVCBs situated in power plants within a specific province in China. The sample dataset encompasses 240 records, encompassing discrete indicators (environmental data) and continuous indicators (mechanical performance, electrical performance, insulation performance), as detailed in Section 2.1. Additionally, the dataset includes four classifications of circuit breaker operating states (Excellent, Fine, Abnormal, Severe).

### 5.2. Validation Method

This paper adopts a 7:3 ratio for partitioning experimental data, specifically utilizing 168 records for training and 72 records for testing. When comparing the evaluation results with the testing data, the study employs receiver operating characteristic (ROC) curves and precision recall (PR) curves to collectively assess the outcomes. Building upon these curve types, the evaluation method employs the area under the ROC Curve (AUC) as a benchmark parameter for assessing the effectiveness of the evaluation. A higher AUC value indicates a more accurate assessment.

To account for uncertainty in the AUC calculation process, we introduce two additional evaluation indicators, the standard error (SE) and confidence interval (CI), with a 95% confidence interval for CI.

### 5.3. Test Result Analysis

#### 5.3.1. Internal Model Test Comparison

**Figure 7:**
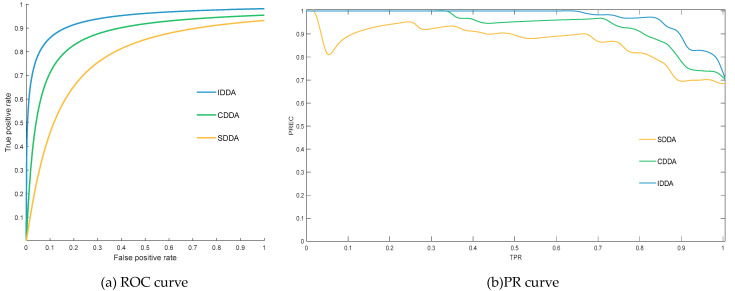
Internal model test results.

To ascertain the effectiveness of the proposed IDDA method, two internal evaluation models are introduced for comparative analysis. The first model is the “CDDA” method, employing a data-driven approach based on CFS to manage discrete indicators, while continuous indicators are processed using a traditional fuzzy inference system. The second model is the “SDDA” method, which solely relies on the OFI system to handle diverse data. The comparative test results for the three evaluation methods are summarized in Table 6 and presented in Figure 7.

As depicted in Table 6, IDDA demonstrates enhanced performance in both evaluation standards, SE and CI, indicative of more reliable and authentic results. Furthermore, Figure 7 reveals that the evaluation method based on IDDA exhibits notably higher AUC values on the ROC and PR curves compared to the CDDA and SDDA models. This suggests superior performance of IDDA.

**Figure 8:**
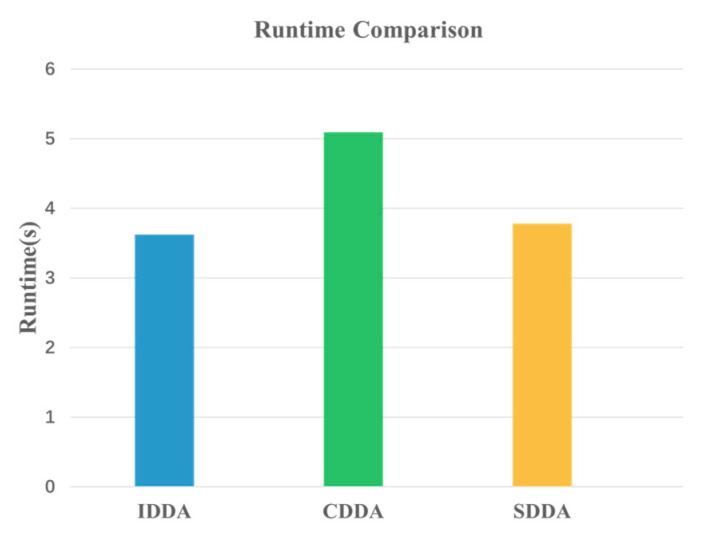
Comparison of runtime for different models.

Additionally, the runtime comparison for each method is depicted in Figure 8. The runtime of the IDDA method closely aligns with the SDDA method, but it is 38.39% less than the runtime of the CDDA method. This disparity can be attributed to CDDA’s reliance on traditional fuzzy inference, necessitating the formulation of a substantial number of fuzzy rules and membership functions. As a result, the execution speed is relatively slow.

#### 5.3.2. External Model Test Comparison

**Figure 9:**
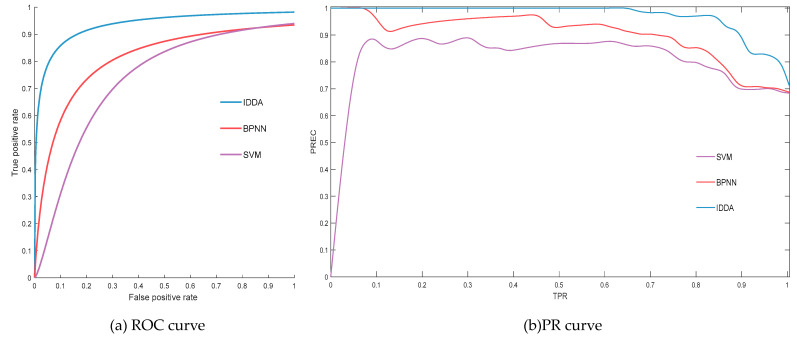
External model test results.

To further affirm the superiority of the IDDA method, we introduce two external models for comparative testing, namely, methods based on BPNN and Support Vector Machine (SVM). ROC and PR curves for each method are plotted, and the SE and CI values for each method are calculated, as summarized in Table 7 and depicted in Figure 9.

From the aforementioned results, it is evident that the proposed IDDA model attains the highest accuracy in state assessment. In terms of both SE and CI evaluation standards, the IDDA method exhibits superiority. Additionally, the AUC of IDDA surpasses that of the BPNN method by 15.77% and the SVM method by 28.23%. In summary, this method outperforms conventional machine learning approaches and yields more satisfactory results.

## 6. Conclusions

The present study introduces a novel approach for assessing the operational condition of SF6 HVCBs leveraging the IDDA framework. The primary contributions of this research can be summarized as follows:

- By integrating both continuous performance metrics and discrete environmental indicators, the evaluation process achieves a heightened level of comprehensiveness, meticulousness, and precision.
- An enhanced methodology for computing diagnostic criteria is proposed, demonstrating efficacy in discerning RP factors. Leveraging the CFS analysis technique allows for an in-depth examination of how fluctuations in environmental variables affect the overall stability of the equipment.
- The implementation of the OFI system for managing continuous performance indicators results in a reduction in membership functions and fuzzy rules. This reduction in complexity enhances evaluation efficiency, thereby accelerating the assessment process.

## Figures and Tables

**Table 1:** Discrete indicators.

Indicator	Factors Included
Weather	Clear, cloudy, overcast, stormy, rainy, snowy
Pollutants	Dust, salt spray, chemical residues, oil contamination
Mechanical Vibration	Equipment operation, construction activities, earthquakes, explosions
External Physical Damage	Bird strikes, vegetation contact, animal chewing, human vandalism
Electromagnetic Interference	Lightning strikes, radio interference, radio frequency interference

**Table 2:** Continuous indicators.

Performance	Indicator	Safety Thresholds
Mechanical	Opening and closing time (ms)	(65,130)
	Opening and closing speed (m/s)	(3.1,5.6)
	Total contact travel (mm)	(190,213)
Electrical	Breaking current value (kA)	(31.5,63)
	Opening coil voltage (V)	$U_{o}=(0.65,1.1)U_{n}$
	Closing coil voltage (V)	$U_{c}=(0.85,1.1)U_{n}$
	Opening coil current (A)	$U_{o}/R$
	Closing coil current (A)	$U_{c}/R$
Insulation	Gas temperature (°C)	(−40,110)
	Gas leakage value (%)	(0.5,1)
	Gas pressure (Pa)	(6.25,7)
	Gas density(kg/m^3^)	(45,50)

**Table 3:** Operational state rating.

Circuit Breaker State	Treatment Approach
Excellent	All indicators encompassed by mechanical, insulation, and electrical performance fall within the normal range.
Fine	Some state indicators exceed the normal range, but generally do not pose a significant impact on personal safety, grid security, or operational safety. Monitoring and attention are required, and immediate action may not be necessary.
Abnormal	Some state indicators surpass the normal range, potentially causing substantial impacts on personal safety, grid security, equipment safety, and economic operations. While the circuit breaker may continue to operate for a brief period, prompt action is necessary.
Severe	Some state indicators severely exceed the normal range, presenting an imminent risk to personal safety, grid security, equipment safety, and economic operations. Immediate action is imperative.

**Table 4:** Rules for the first layer of fuzzy inference.

If One Input of the First Layer Is	And Another Input of the First Layer Is	Then the Output Is	The Weight Is
Seldom	Seldom	Very High	0.89
Seldom	Occasionally	Very High	0.89
Seldom	Generally	Elevated	0.58
Seldom	Usually	Very High	0.89
Occasionally	Seldom	Elevated	0.58
Occasionally	Occasionally	Elevated	0.58
Occasionally	Generally	Moderate	0.33
Occasionally	Usually	Elevated	0.58
Generally	Seldom	Moderate	0.33
Generally	Occasionally	Low	0.15
Generally	Generally	Elevated	0.58
Generally	Usually	Elevated	0.58
Usually	Seldom	Moderate	0.33
Usually	Occasionally	Low	0.15
Usually	Generally	Low	0.15
Usually	Usually	Very High	0.89

**Table 5:** Rules for the remaining layers of fuzzy inference.

If the Input of the Next Layer Is	And the Output of Previous Layer Is	Then the Output Is	The Weight Is
Seldom	Low	Moderate	0.33
Seldom	Moderate	Elevated	0.58
Seldom	Elevated	Very High	0.89
Seldom	Very High	Very High	0.89
Occasionally	Low	Moderate	0.33
Occasionally	Moderate	Elevated	0.58
Occasionally	Elevated	Elevated	0.58
Occasionally	Very High	Very High	0.89
Generally	Low	Low	0.15
Generally	Moderate	Moderate	0.33
Generally	Elevated	Moderate	0.33
Generally	Very High	Elevated	0.58
Usually	Low	Low	0.15
Usually	Moderate	Low	0.15
Usually	Elevated	Moderate	0.33
Usually	Very High	Elevated	0.58

**Table 6:** Data for evaluation standards of internal models.

	Evaluation Standards	SE	CI
Model			
IDDA		0.02200	0.89042–0.97664
CDDA		0.03290	0.79794–0.92691
SDDA		0.04361	0.68043–0.85138

**Table 7:** Data for external model test results.

	Evaluation Standards	SE	CI
Model			
IDDA		0.02200	0.89042–0.97664
BPNN		0.03956	0.72885–0.88393
SVM		0.04689	0.63609–0.81989

## Data Availability

The raw data supporting the conclusions of this article will be made available by the authors upon request.
